# Insight into the Impact of Wide Bandgap Transparent Conducting Oxide on the Performance of Thin Film Solar Cells

**DOI:** 10.1002/smll.74042

**Published:** 2026-06-09

**Authors:** Youseong Park, Jun Sung Jang, Dhanaji B. Malavekar, Hojun Choi, Do Hyun Kim, Dong Hyun Kang, Donghoon Song, Seung Wook Shin, Jin Hyeok Kim

**Affiliations:** ^1^ Department of Materials Science and Engineering and Optoelectronics Convergence Research Center Chonnam National University Gwangju Republic of Korea; ^2^ Department of Chemical Engineering Sunchon National University Suncheon Republic of Korea; ^3^ Future Agricultural Research Division Rural Research Institute Korea Rural Community Corporation Ansan‐si Gyeonggi‐do Republic of Korea

**Keywords:** electrical properties, thin‐film solar cells, transparent conducting oxide, wide bandgap energy, ZnO‐based compounds

## Abstract

The excellent optoelectronic properties of transparent conducting oxides (TCOs) are critical for achieving highly efficient thin‐film solar cells (TFSCs). In this regard, Mg‐ and group III‐codoped ZnO‐based compounds are highly promising TCO materials owing to their wide optical spectra, high transmittance, and electrical properties. However, detailed investigations of TFSC applications remain elusive. In this study, we systematically investigated the detailed relationship between various electrical properties (i.e., carrier concentrations and mobility conditions) and the device parameters for kesterite‐based TFSCs. In particular, a quantitative analysis of scattering, detailed Eg‐widening mechanisms in TCOs, and their effects on the quantitative deconvolution of series resistance and TFSC performance is discussed. As a result, the photocurrent densities and fill factors in TFSCs are strongly related to the electrical properties of the TCO, whereas the open‐circuit voltages remain constant regardless of the TCO material. Among the investigated TCO materials, the Mg and Ga‐codoped ZnO TCO layer exhibits favorable band alignment and high transmittance compared with the other layers, significantly enhancing charge transport and suppressing recombination at the interface in TFSC devices. These findings offer new insights into the fundamental impact of wide‐optical‐bandgap energy TCOs on charge transport, interfacial recombination properties, and overall device performance in inorganic‐based TFSC devices.

## Graphical abstract

Mg‐ and Ga‐codoped ZnO has emerged as an effective wide‐bandgap transparent conducting oxide for kesterite thin‐film solar cells, combining high transmittance with favorable electrical properties. By enabling near‐flat band alignment, reduced defect‐assisted recombination, and improved electron transport, Mg‐ and Ga‐codoped ZnO enhances the photocurrent density and fill factor, thereby delivering superior performance in CZTSSe solar cells compared to conventional ZnO‐based TCOs.

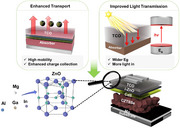

## 1 Introduction

Transparent conducting oxides (TCO) are indispensable materials for optoelectronic devices such as displays, sensors, and photodetectors [1, 2, 3]. In particular, they have been widely employed as window layers in multilayered structures of inorganic thin‐film solar cells (TFSCs) such as Cu(In_1‐x_,Ga_x_)Se_2_ (CIGS) [4], Sb_2_(S,Se)_3_ [5] and Cu_2_ZnSn(S,Se)_4_ (CZTSSe) [6]. By contrast, an ideal TCO should possess a high transmittance along with a sufficiently wide optical bandgap energy (*E*_g_) to suppress parasitic absorption near short wavelengths. A low optical transmittance or narrow *E*_g_ in a TCO leads to front‐side optical losses in the blue/visible wavelength region [7], which in turn restricts the achievable short‐circuit current density (*J*_sc_) [8]. Optimizing the device performance of TFSCs requires a synergy of favorable electrical and interfacial properties. Specifically, low sheet resistance, high carrier concentration, superior mobility, and precise band alignment are pivotal for minimizing the series resistance (*R*_s_) and suppressing barrier formation, both of which directly affect the fill factor (*FF*) [9, 10]. Furthermore, a smooth surface morphology and work function tailored to the TCO properties are critical factors for achieving outstanding device performance in TFSCs because they reduce interfacial defects and facilitate favorable band alignment.

Indium tin oxide (ITO) is the most widely used TCO material in TFSCs owing to its excellent optoelectronic properties, including low resistivity, high optical transmittance, and a suitable *E*_g_ [11, 12]. However, the scarcity and cost of indium hinder the cost‐effectiveness and large‐scale commercialization of TFSC devices. Moreover, ITO suffers from process‐related drawbacks when integrated into practical TFSC devices, including thermal instability and damage to the underlayer (i.e., chemical bath‐deposited CdS) under plasma deposition conditions. These issues can significantly deteriorate optoelectronic device performance, underscoring the necessity for viable alternatives to ITO [13, 14]. Among the explored alternatives, ZnO‐based compounds doped with group‐III elements, such as Al, In, and Ga, have attracted considerable attention as promising materials for alternative TCO [15, 16, 17, 18]. These materials exhibit high commercial potential owing to their low cost, nontoxicity, excellent chemical and thermal stability, wide *E*_g_ (>3.4 eV), and low electrical resistivity (below ∼10^−4^ Ω cm) [19]. Recently, there has been increasing interest in developing quaternary ZnO‐based TCOs by introducing an additional dopant into ternary systems to improve their optical and electrical properties. Previous investigations have demonstrated that codoping ZnO with two or more elements can yield higher carrier mobility and lower resistivity than single‐element‐doped ZnO.

In particular, doping Mg into ZnO‐based ternary TCOs (i.e., Al‐doped ZnO (AZO) or Ga‐doped ZnO(GZO)) significantly enhances not only the electrical characteristics but also the transmittance in the visible region and *E*_g_ [20, 21]. The implementation of quaternary TCOs with excellent optoelectronic properties as window layers in TFSC enables improved charge carrier collection in the short‐wavelength region of the external quantum efficiency (EQE) spectrum owing to a wider *E*_g_ and promotes a flattened band profile through favorable conduction band offset (CBO) alignment [7]. In addition, the lower resistivity and higher mobility of TCOs mitigate interfacial recombination, underscoring the importance of optimizing the window layer in TFSC devices. In this regard, our group investigated the optoelectronic properties of ZnO‐based TCO materials by introducing various doping elements, doping concentrations, and deposition conditions. The excellent electrical and optical properties (i.e., <10 Ω/sq and >3.8 eV) were achieved in Mg‐ and Ga‐doped ZnO (MGZO), Mg‐ and Al‐doped ZnO (MAZO), and Mg‐ and In‐doped ZnO (MIZO) systems [22, 23]. ZnO‐based systems codoped with Mg and Ga (MGZO), and Mg and Al (MAZO) have demonstrated excellent electrical and optical properties, characterized by low sheet resistance (<10 Ω/sq) and wide optical bandgaps (>3.7 eV) [22]. Furthermore, TFSCs employing MGZO as a window layer exhibited a notable enhancement in the EQE spectrum by approximately 10% at wavelengths below 420 nm, leading to improved device performance compared to those employing AZO [24].

While our previous work primarily focused on the material synthesis and basic optoelectronic properties of the MGZO system to establish its proof‐of‐concept application for reducing short‐wavelength parasitic absorption, a comprehensive understanding of how different codopants fundamentally alter the device physics remains largely unexplored. In the current study, we move significantly beyond the fundamental characterization of materials to systematically investigate the underlying interfacial physics and charge transport mechanisms using a series of Mg and Group III (Al, Ga, or In) codoped ZnO TCOs. The central advancement of this work lies in the direct correlation of the tailored properties of these TCOs with the intricate device physics of kesterite TFSCs. Specifically, by employing ultraviolet photoelectron spectroscopy (UPS) and capacitance (C‐V) analyses, we demonstrated how the modulated Fermi levels and conduction band minimum (CBM) of the respective TCOs directly dictate the heterointerface band alignment and physically govern the depletion width (W_d_) extending into the absorber. Furthermore, we established a multifaceted correlation demonstrating how the diverse optical and electrical parameters of the TCO layers, such as carrier concentration, mobility, and E_g_, individually and synergistically affect the specific device characteristics of TFSCs. Ultimately, this comprehensive study provides new scientific insights into the buried interfacial physics and offers universal design guidelines for optimizing the window layer to maximize the efficiency of multilayer TFSCs, explicitly distinguishing it from prior phenomenological reports.

**FIGURE 1:**
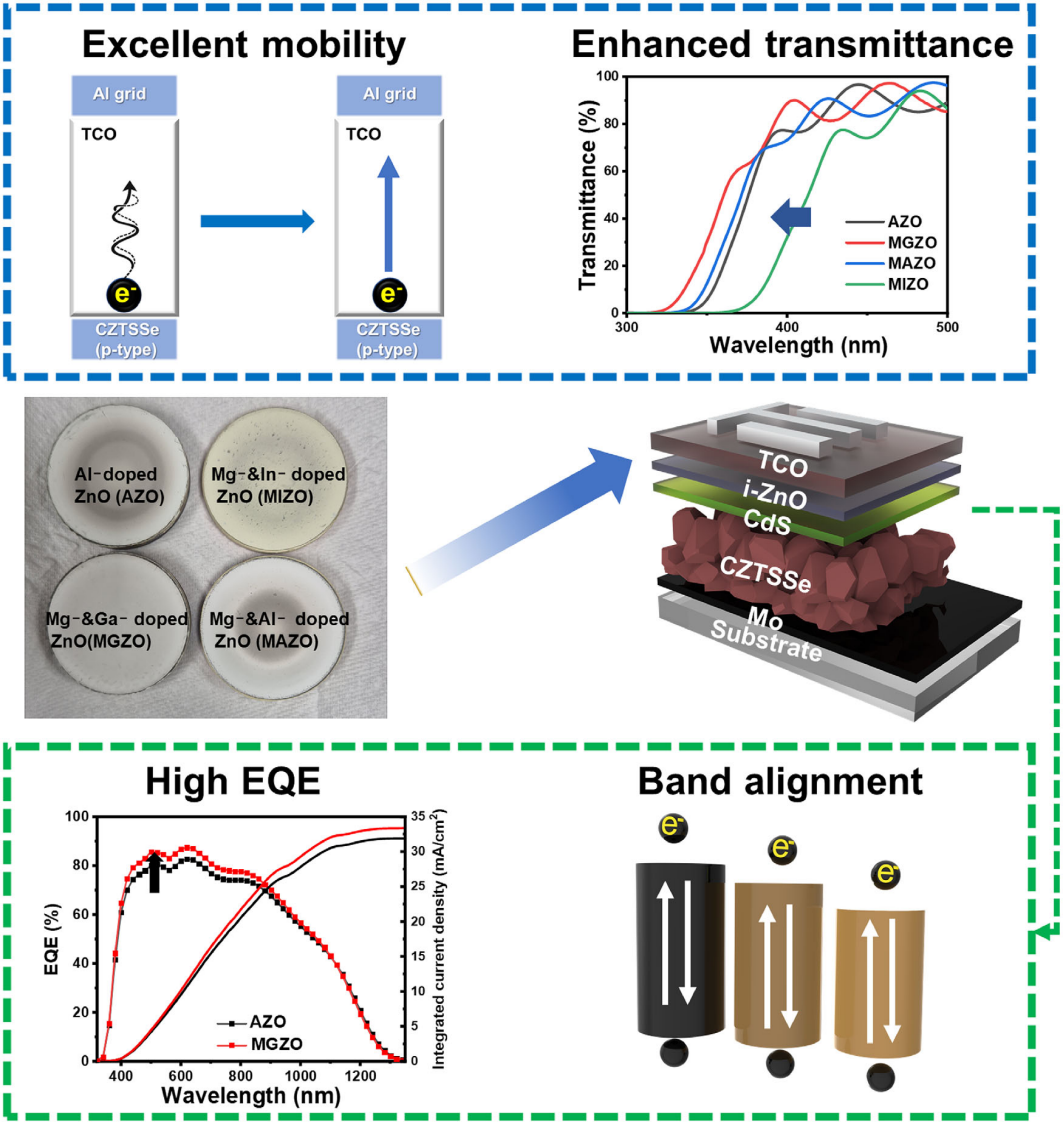
Schematic illustration of the wide *E*_g_ TCO with excellent electrical properties and corresponding device parameters for CZTSSe TFSC under different TCO layer conditions.

In this study, we synthesized wide *E*_g_ ZnO‐based TCOs with tailored electrical properties (i.e., high carrier concentrations and mobilities) while maintaining a high optical transmittance by incorporating Mg into In‐, Ga‐, or Al‐doped ZnO. We mainly discuss the quantitative analysis of scattering and detailed E_g_ widening mechanisms for wide *E*_g_ ZnO‐based TCOs. These materials were subsequently integrated into the kesterite TFSCs, as illustrated in Figure 1. We systematically investigated the relationships between the electrical properties of TCOs and the device parameters of kesterite TFSCs by mapping the photovoltaic metrics, interfacial band alignment, and junction quality. As a result, using MGZO as the TCO layer, we achieved an increase in the power conversion efficiency (PCE) from 10.18% to 11.30%, which is attributed to improved charge transport arising from favorable band alignment and enhanced junction quality compared with those of the other TCOs.

## 2 Results and Discussion

### 2.1 Optical Properties of Codoped ZnO‐Based TCO Thin Films

**FIGURE 2:**
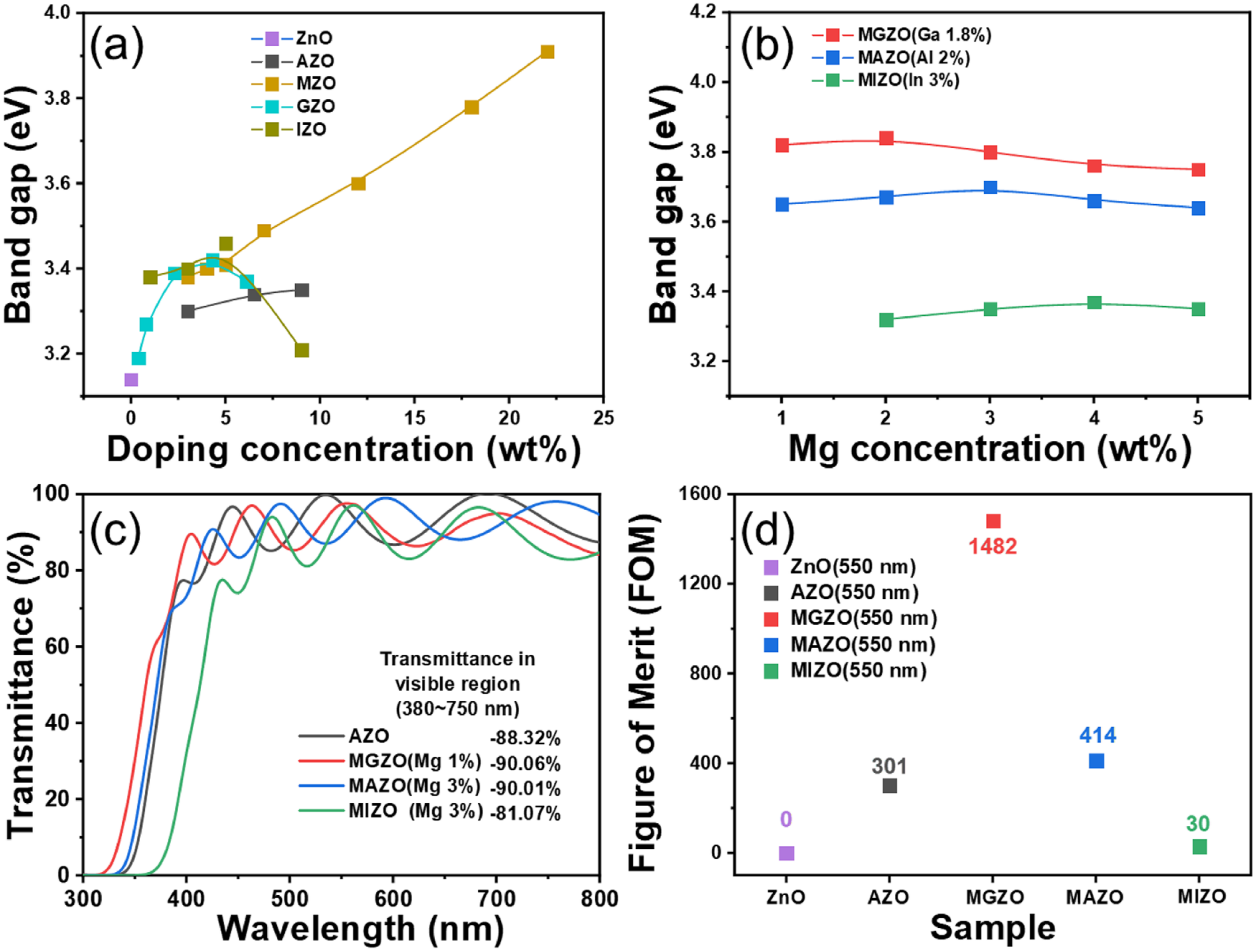
(a) *E*_g_ from ZnO‐based compounds in the previously reported literatures [25, 26, 27, 28, 29, 30]. The detailed information is presented in Table S1 in the Supporting Information. (b) *E*_g_ obtained from this study, (c) transmittance, and (d) FOM values of wide *E*_g_ ZnO‐based TCO with different doping elements and concentrations. The detailed calculation process of FOM values is presented in the Supporting Information.

The influence of group III elements (Al, Ga, or In) and Mg content on *E*_g_ was evaluated using recently published reports [25, 26, 27, 28, 29, 30, 31] on ZnO crystal systems, as shown in Figure 2a. For AZO, the values for *E*_g_ are linearly widened with increasing Al concentration. By contrast, Ga‐doped ZnO (GZO) and In‐doped ZnO (IZO) exhibited pronounced *E*_g_ variations with increasing dopant concentration up to 5 wt.%, after which the *E*_g_ decreased at higher concentrations. The bandgap differences between ZnO and GZO or IZO reach approximately 0.2−0.3 eV at 5 wt.% dopant concentration. These observations indicate that the incorporation of group III elements alone offers limited capability to widen *E*_g_ in the ZnO crystal structure. By contrast, Mg‐doped ZnO (MZO) shows a sharp linear *E*_g_ expansion up to ∼25 wt.% Mg, indicating that Mg plays a decisive role in *E*_g_ enlargement in ZnO‐based TCO. To directly evaluate how Mg incorporation into ternary ZnO affects *E*_g_, we prepared quaternary ZnO thin films by introducing Mg and group III elements into the ZnO crystal structure. This study considered Mg contents of only up to 5%, owing to the possibility of crystal transformation from a hexagonal to a cubic phase under high Mg concentrations [32].

The results and the corresponding discussion regarding the crystal structure, morphology, thickness, and surface roughness of AZO, MGZO, MAZO, and MIZO are provided in Note S1 Supporting Information. As shown in Figure 2b, with increasing Mg content, *E*_g_ increased sequentially in the order MIZO<MAZO<MGZO. The increase in *E*_g_ is primarily ascribed to the reduced electron affinity and elevation of the CBM resulting from Mg incorporation into the ZnO crystal system [33]. Figure 2c shows the transmittance of the ZnO‐based compounds with different dopants in the UV–vis wavelength region. The optical and electrical properties of ZnO‐based TCOs are significantly influenced by the doping concentration [22, 23, 24] (See Figure S1). All the TCO thin films exhibited average transmittance values exceeding 80% in the visible region. In particular, MGZO and MAZO exhibited average transmittance values exceeding 90%, which were higher than those of AZO (88.32%) and MIZO (81.07%). The higher transmittance can be attributed mainly to the reduced scattering and defect‐related parasitic absorption arising from their superior crystallinity (See Figure S2 and Note S1) and/or a lower surface roughness (See Figure S3 and Note S2 and Table S2) [9, 34]. Notably, the absorption edge shifts toward shorter wavelengths in the following order: MGZO > MAZO > AZO > MIZO. This indicates that even short‐wavelength light can be efficiently transmitted. Additional optical properties, including photon energy vs. (*ahv*)^2^, reflectance, and absorptance, are shown and discussed in Figure S4 and Note S3. Figure 2d shows the figure of merit (FOM) of the ZnO‐based TCO with different dopants. The FOM, proposed by Gruner as a performance index for transparent electrodes [35], was calculated from the transmittance and sheet resistance (*R*_sh_). Detailed equations and calculation procedures are provided in the Supporting Information. The FOM enables an intuitive comparison of the transparent electrode performance [36]. All the ZnO‐based TCOs exhibited higher FOM values than pristine ZnO, which can be attributed to the substantial improvements in transmittance and *R*_sh_ upon the incorporation of Mg and group‐III elements. MGZO showed the highest FOM value (1482), indicating superior overall optical and electrical properties among the investigated samples.

### 2.2 Electrical Properties of ZnO‐Based TCOs

**FIGURE 3:**
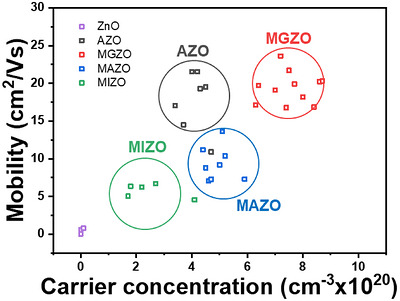
Mobility vs carrier concentrations of wide *E*_g_ ZnO‐based TCO thin films, including ZnO, AZO, MGZO, MAZO, and MIZO, respectively. The data were obtained from different deposition conditions and doping concentrations.

The electrical properties of undoped ZnO‐, AZO‐, and quaternary‐ZnO‐based TCO thin films were systematically investigated using Hall measurements at room temperature, as shown in Figure 3. The measured carrier concentrations and mobilities provide insight into the role of dopant elements and structural quality in the charge transport behavior. Undoped ZnO exhibited relatively poor electrical properties (i.e., electron mobility in the range of 0.04−1 cm^2^/Vs), indicating limited carrier transport properties in multistructured optoelectronic devices. By contrast, all doped ZnO‐based TCO thin films exhibited an improvement in mobility of over 5 cm^2^/Vs, confirming that donor doping enhances electronic transport in the ZnO crystal system. Among the quaternary ZnO TCOs, MIZO showed inferior electrical properties (i.e., <4 cm^2^/Vs of mobility) compared to those of AZO, MAZO, and MGZO. Although these films exhibited a relatively low surface roughness (Figure S3 and Table S2), the limited enhancement in the mobility and carrier concentration may be attributed to poor crystallinity and the absence of a well‐defined *c*‐axis preferred orientation, as observed in the XRD patterns (Figure S2). These factors can increase carrier scattering and limit the effective charge transport [22]. MAZO thin films exhibit higher carrier concentrations than AZO thin films; however, their mobilities are lower. This behavior is primarily attributed to ionized impurity scattering arising from heavy carrier concentrations, which distort the ZnO lattice and hinder carrier mobility [37]. The higher surface roughness observed for MAZO (Table S2) further enhances carrier scattering at the surface and grain boundaries, ultimately contributing to reduced mobility. However, the MGZO thin film showed the best electrical properties (i.e., 8.78 × 10^20^ cm^−3^ of carrier concentration and a mobility of 24 cm^2^/Vs). Unlike the AZO and MAZO thin films, which showed an inverse relationship between mobility and carrier concentration, the MGZO thin films exhibited a proportional enhancement in both parameters. This behavior deviates from the commonly observed trade‐off under heavy doping conditions, where an increased carrier concentration typically leads to mobility reduction owing to enhanced ionized‐impurity scattering [37]. The physical origin of this distinctive electrical property in MGZO can be rigorously elucidated using Matthiessen's rule, supported by additional Hall measurements at both room temperature (300 K) and low temperature (77 K) under liquid‐nitrogen conditions (See Figure S5). The negligible variation in both carrier concentration and mobility at 77 and 300 K indicates a typical degenerate semiconductor behavior, implying that temperature‐dependent phonon scattering (µ_*phonon*_) plays a minimal role. Thus, the total mobility is predominantly governed by ionized impurity (µ_*ii*_), grain boundary (µ_*gb*_), and neutral impurity (µ_*ni*_) scatterings. Although a higher carrier concentration inherently intensifies the concurrent enhancement of the mobility in MGZO, it originates from the drastic suppression of other dominant scattering mechanisms. Specifically, MGZO's distinctive electrical properties also are strongly related to its superior crystallographic characteristics relative to the other materials (See Figure S2a).

Normally, differences in the ionic radii of dopants can introduce additional lattice strain into the ZnO crystal system. The ionic radii of Mg^2+^ (0.072 nm) and Ga^3+^ (0.062 nm) are much closer to that of Zn^2+^ (0.074 nm) than that of Al^3+^ (0.053 nm) [38]. This ionic size matching enables the Mg and Ga dopants to more effectively occupy the Zn lattice sites with minimal distortion [39]. This structural stability substantially inhibits the formation of compensating defects and neutral defect clusters, thereby minimizing µ_*ni*_. Furthermore, the superior crystallinity of MGZO intrinsically reduces the density of the grain boundaries. Combined with its exceptionally high carrier concentration, the depletion barrier width at the remaining grain boundaries is heavily narrowed, allowing carriers to transport easily via tunneling [40, 41], which drastically suppresses µ_*gb*_. Consequently, the profound reduction in both µ_*ni*_ and µ_*gb*_ within the structurally relaxed MGZO lattice outweighs the conventional increase in µ_*ii*_, enabling the simultaneous improvement of carrier concentration and mobility. The MGZO thin films exhibited a dense and smooth surface morphology, as evidenced by their lower surface roughness compared to those of MAZO and AZO (See Figure S3 and Table S2). Furthermore, the high crystallinity and larger grain sizes of MGZO significantly reduced the trap densities at the grain boundaries, allowing electrons to move more easily and enabling the simultaneous enhancement of effective carrier transfer and collection.

### 2.3 Correlation Between Electrical Properties for TCO and Device Performances

**FIGURE 4:**
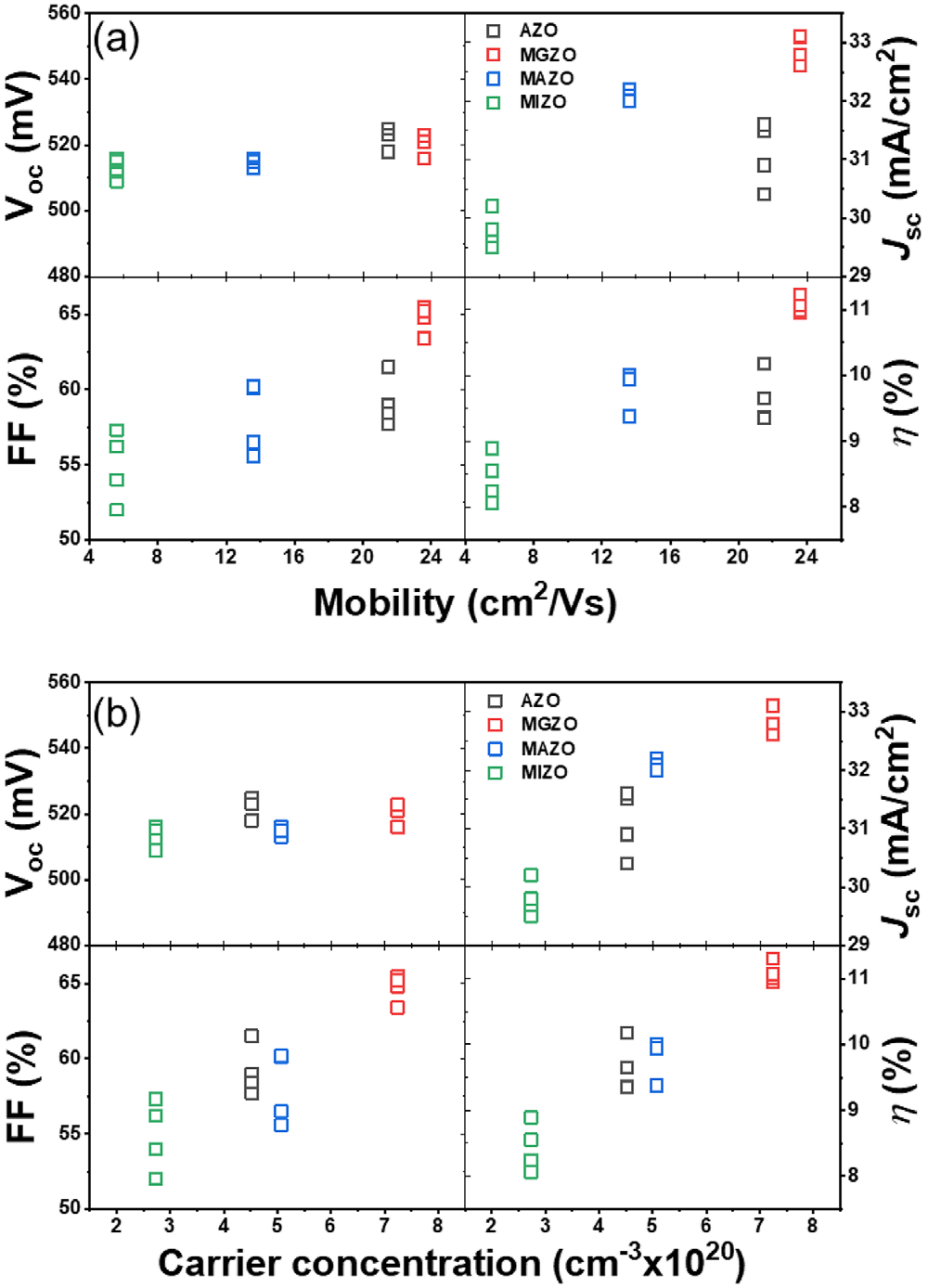
Relationships between electrical properties, including (a) mobility and (b) carrier concentration of ZnO‐based wide *E*_g_ TCOs and device parameters in kesterite TFSCs.

Figure 4 shows the correlations between the electrical properties, including the mobility and carrier concentration of ZnO‐based wide *E*_g_ TCOs, and the device parameters of the kesterite TFSCs. The crystallographic and microstructural analyses of the kesterite absorber layer are shown in Figures S6–S8 and Note S4 the corresponding device parameters are summarized in Table 1. All the TFSC devices exhibited V_oc_ values exceeding 500 mV, regardless of the electrical properties of the TCO layers. V_oc_ was predominantly governed by nonradiative recombination induced by the high defect density in the CZTSSe absorber bulk and at the CZTSSe/CdS interface [42]. Although the CBO at the *i*‐ZnO/TCO interface varied from −0.04 to −0.15 eV, this interface forms an *n*‐*n^+^* isotype heterojunction located far from the primary *p‐n* junction, where minority carrier (*i.e*., hole) recombination mainly occurs. Furthermore, such a small negative CBO does not act as a significant barrier to the photogenerated electrons, nor does it induce massive interface recombination, which degrades V_oc_ [43, 44, 45]. By contrast, the influence of the TCO layer on V_oc_ is relatively limited, and it more strongly affects *J*_sc_ and *FF*, primarily through optical transmittance or absorption losses and electrical losses associated with the series resistance and contact properties [46].

**TABLE 1:** Average and best device performances of kesterite‐based TFSCs with AZO, MGZO, MAZO, MIZO TCOs as window layers. Data was obtained from 4 ∼ 6 solar cells.

Sample codes		V_oc_ (mV)	*J*_sc_ (mA/cm^2^)	*FF* (%)	PCE (%)
AZO	Avg.	522 ± 4	31.1 ± 2	59.1 ± 2.4	9.63 ± 0.55
	Best	525	31.6	61.5	10.18
MGZO	Avg.	518 ± 5	32.8 ± 0.4	64.9 ± 1.5	11.02 ± 0.28
	Best	523	33.1	65.7	11.30
MAZO	Avg.	515 ± 2	32.1 ± 0.1	58.1 ± 3.4	9.77 ± 0.39
	Best	516	32.2	60.2	10.00
MIZO	Avg.	513 ± 4	29.8 ± 0.4	54.8 ± 2.8	8.43 ± 0.46
	Best	516	30.2	57.3	8.89

As shown in Figure 4a, the electrical mobility and device parameters (i.e., *J*_sc_, *FF*, and PCE (*η*)) exhibit nearly linear improvement. In the multilayered architecture of kesterite TFSCs, charge‐carrier transport is significantly influenced by multiple loss mechanisms, including interfacial defects, defect clusters, impurities, recombination, and resistive voltage drop [47, 48, 49]. The higher mobility of the TCO layer in such multilayered devices reduces the resistive losses along the transport path and suppresses the voltage drop under a high current density and voltage bias, thereby improving the *FF* [50, 51]. Consequently, the kesterite TFSC fabricated with MGZO as the TCO layer exhibited a higher *FF* (65.7%) than that fabricated with MIZO (57.3%). The carrier concentration shows a linearly proportional relationship with *J*_sc_ and *η* (Figure 4b). TCO thin films with higher carrier concentrations provide higher *J*_sc_ values in TFSCs. In heavily doped semiconductors, the apparent optical E_g_ (Δ*E_opt_*) is determined by the interplay between the Burstein‐Moss (BM) shift (Δ*E_BM_*) and E_g_ renormalization (Δ*E_BGR_*) induced by many‐body effects, expressed as Δ *E_opt_* = *E*_*g*0_  +  Δ*E_BM_* − Δ*E_BGR_*, (where *E*_*g*0_ is the fundamental E_g_ of the host lattice). As the carrier concentration (n) increases, the lowest states in the conduction band are filled, leading to an optical E_g_ widening (Δ*E_BM_*), following the relation, ΔE_BM_ = $\frac{\hbar^{2}}{2m^{*}}x\;{\left(3\pi^{2}n\right)}^{\frac{2}{3}}$. Conversely, the high density of charge carriers induces electron‐electron and electron‐ion interactions, causing a E_g_ narrowing (Δ*E_BGR_*) proportional to $n^{\frac{1}{3}}$ [52, 53]. In the case of MGZO, despite the expected Δ*E_BGR_* resulting from its exceptionally high carrier concentration (>7.0 × 10^20^ cm^−3^), the material still exhibited the widest Δ*E_opt_* of 3.82 eV. This indicates that a substantial Δ*E_BM_* significantly outweighs the Δ*E_BGR_* effect (See Table S3). Furthermore, the intrinsic widening of *E*_*g*0_ due to the structural alloying of Mg^2+^ into the ZnO lattice also contributes to this wide Δ*E*_opt_. Consequently, this net expansion of the optical E_g_ reduces the parasitic absorption in the short‐wavelength region, thereby improving *J_sc_* [54]. Overall, the TFSC device efficiencies followed the order MIZO (8.43%) < AZO (9.63%) < MAZO (9.77%) < MGZO (11.02%). Interestingly, despite its relatively poor electrical properties, the MAZO‐based TFSC delivered a device performance comparable to that of AZO. This phenomenon suggests that the device parameters are strongly related to both electrical and optical properties. The MAZO thin films exhibited better transmittance in the visible wavelength region and a higher FOM value, as shown in Figure 2, indicating that more light was irradiated onto the TFSCs. Additionally, a favorable band alignment (i.e., CBO) can contribute to improved device performance, which is discussed in the following section.

### 2.4 Band Alignment of Kesterite TFSCs With Various ZnO‐Based TCOs

**FIGURE 5:**
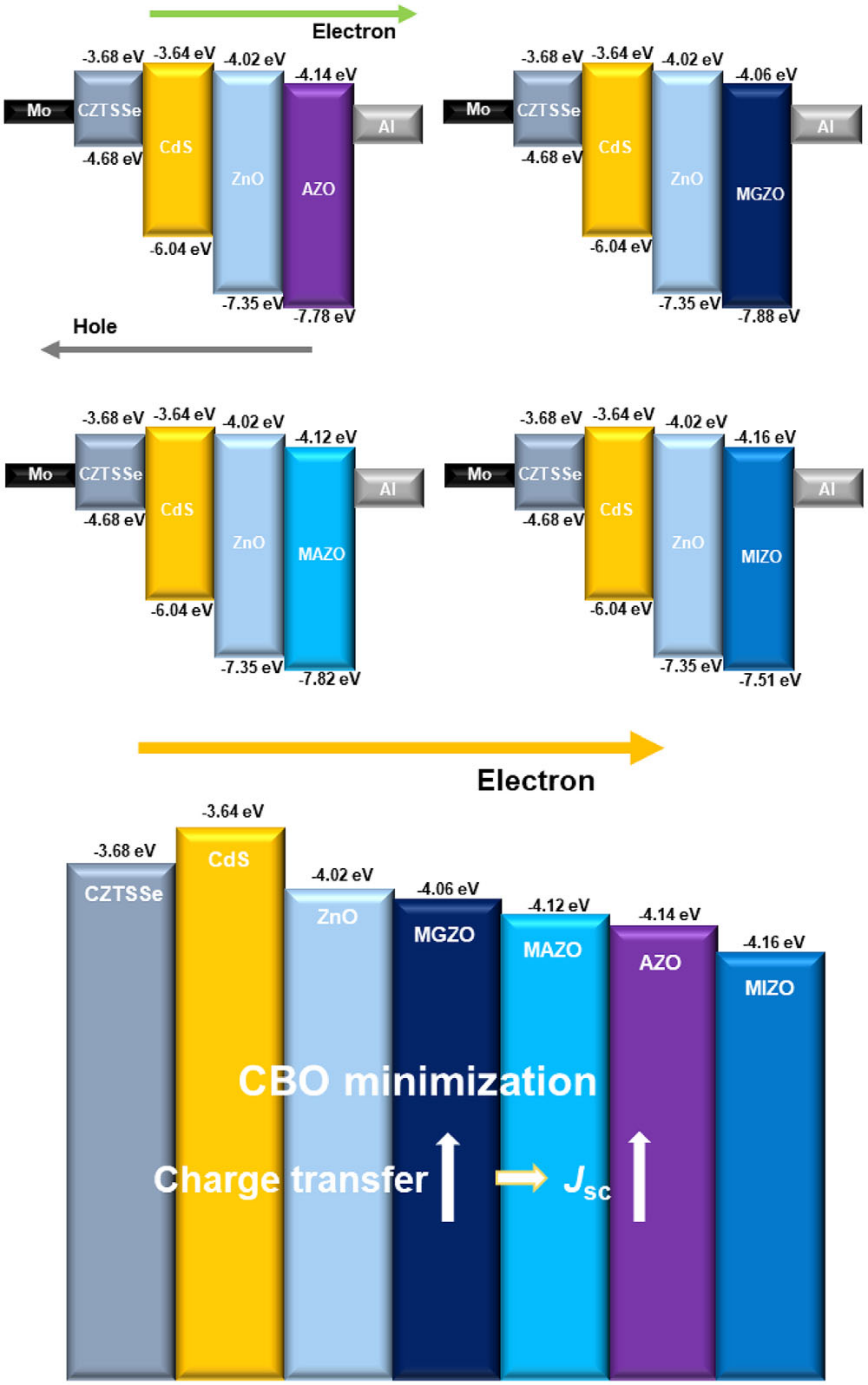
Energy band schematics of the kesterite TFSCs with ZnO‐based wide *E*_g_ TCOs. Detailed information on band alignments is provided in Figure S9 and Table S3.

Figure 5 shows the band alignments of kesterite TFSCs incorporating various ZnO‐based wide *E*_g_ TCOs as window layers. A schematic was constructed based on calculations derived from ultraviolet photoelectron spectroscopy (UPS) spectra (See Figure S9), which provides direct insight into the carrier transport mechanisms at the interfaces of the full device and constituent layers. The calculation procedure and band alignment construction were performed as described previously [45, 55]. The secondary electron cutoff and valence band maximum (VBM) were determined using the linear extrapolation method, in which the intersection between the baseline and the leading‐edge slope was precisely identified [56]. To ensure repeatability, measurements were conducted at multiple points for each sample, and the average values were used for band offset calculations. Considering the instrumental resolution of the UPS system (±0.1 eV) and the uncertainty in optical gap estimation, the calculated CBO values carry an estimated error propagation of approximately ± 0.1 eV. The *E*_g_ values of CdS, TCO, and kesterite were obtained from the UV–vis and EQE spectra. Detailed values of the CBM, CBO, and VBM are summarized in Table 2. In kesterite TFSCs, the photoexcited electrons are extracted along the pathway; absorber → CdS → *i*‐ZnO → TCO → metal grid. The CBO at each junction plays a crucial role in governing the device performance; values close to zero facilitate electron transfer to the electrode and enhance photocurrent collection, whereas large positive offsets act as barriers or traps, thereby limiting carrier injection [7, 55, 57]. Our results demonstrate that Mg‐incorporated ZnO‐based TCOs exhibit higher CBM positions than AZO or undoped ZnO TCOs [58]. Consequently, the CBO at the *i*‐ZnO/TCO interface decreases systematically in the order MIZO (−0.15 eV) > AZO (−0.13 eV) > MAZO (−0.10 eV) > MGZO (−0.04 eV). Notably, MGZO forms a nearly flat conduction band alignment with *i*‐ZnO, characterized by an exceptionally small CBO value of −0.04 eV, which is highly favorable for electron transport [59]. In this regard, the similar CBM positions of *i*‐ZnO/ZnO‐based TCOs contribute to the improved *J*_sc_ values [60]. This trend suggests that the Mg incorporation strategy enables effective tuning of the current transport through the TCO by modulating the *E*_g_ of the TCO, thereby achieving a suitable band alignment. These findings demonstrate that band alignment engineering in kesterite TFSCs is possible by adjusting the window layer using various quaternary‐ZnO‐based TCO materials.

**TABLE 2:** CBM, VBM, CBO values corresponding to the AZO, MGZO, MAZO, and MIZO samples.

Sample	CBM	VBM	CBO
AZO	−4.14	−7.78	−0.12
MGZO	−4.06	−7.88	−0.04
MAZO	−4.12	−7.82	−0.10
MIZO	−4.16	−7.51	−0.14

### 2.5 Device Performance and Recombination Under Dark Conditions

**FIGURE 6:**
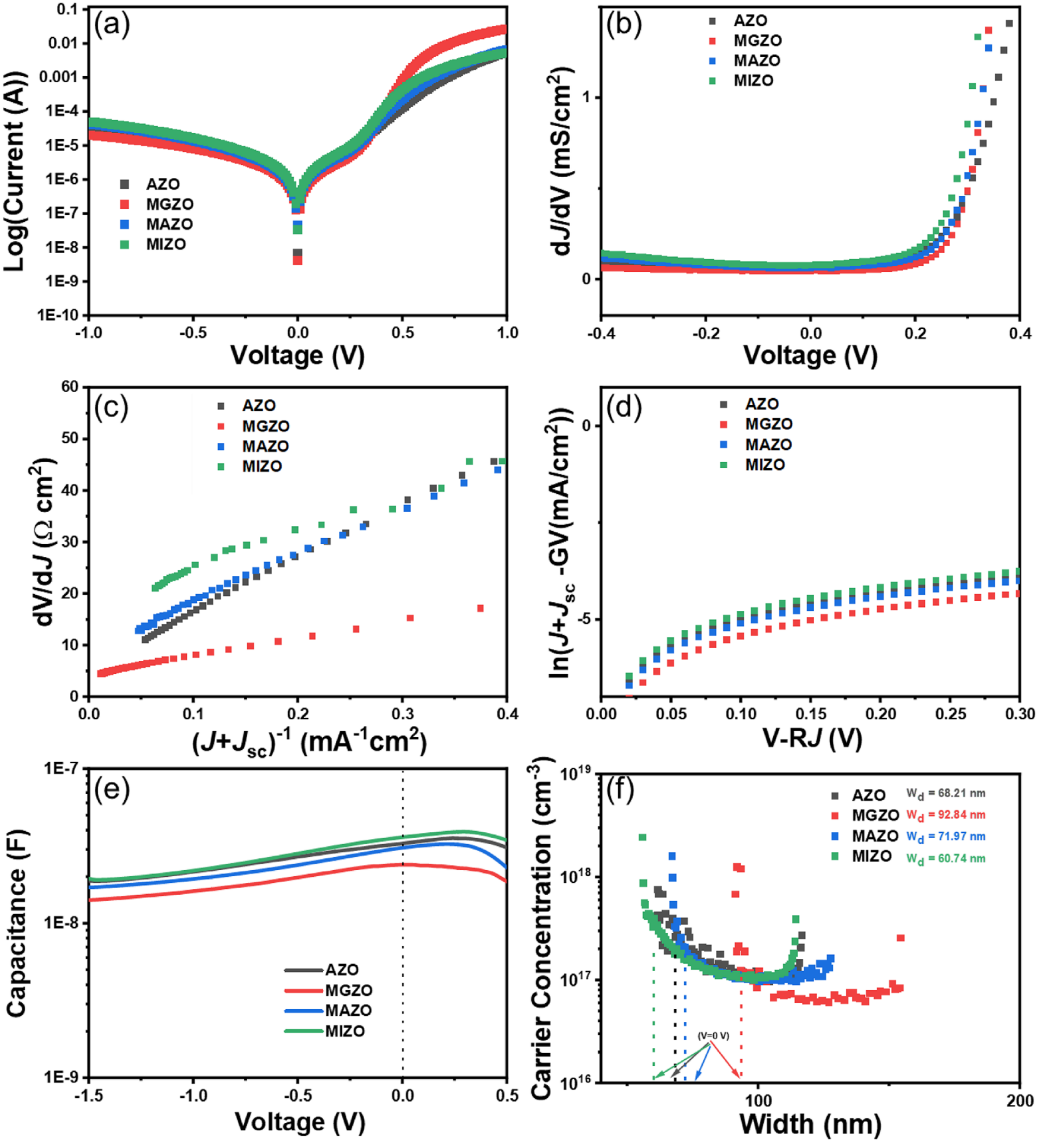
(a) Dark *I–V* curves, (b) d*J*/dV vs. V, (c) dV/dJ vs. (J + J_sc_)^−1^, and (d) ln (*J*+J_sc_ ‐GV vs. V‐R*J*), (e) C‐V plots, and (f) N_a_ *vs*. W_d_ of kesterite TFSCs with various ZnO‐based wide *E*_g_ TCOs.

The influence of various TCOs as window layers in TFSCs was evaluated using dark *J*‐V curves to identify the carrier recombination mechanisms. The W_d_ and acceptor concentration (N_a_) were estimated from the C‐V plots to assess *p‐n* junction quality. The equations and detailed calculation procedures for *R*_s_, saturation current density (*J*_0_), and shunt conductance (G_sh_) obtained from the dark *J*‐V curves are provided in Supporting Information. The detailed values of these parameters for all devices are summarized in Table 3. Figure 6a–d shows the dark *J–V* curves and corresponding plots for kesterite TFSCs with various ZnO‐based wide *E*_g_ TCOs. The device with MGZO exhibited a much lower G_sh_ (0.0437 mS/cm^2^) than the other devices, indicating that MGZO suppressed the shunt paths and reduced the leakage current more effectively than the other TCOs in kesterite TFSCs [61]. As shown in Figure 6c, the *R*_s_ values followed the order MGZO (3.07 Ω cm) < MAZO (8.04 Ω cm < AZO (5.88 Ω cm) < MIZO (15.01 Ω cm). The relatively low *R*_s_ values of the MGZO‐based TFSC are consistent with their excellent electrical properties. Under dark conditions, *R*_s_ was predominantly governed by the transport properties of the TCO layer [62]. Although MAZO possesses a higher carrier concentration than AZO, severe ionized impurity scattering drastically reduces the carrier mobility, fundamentally limiting its total conductivity and resulting in a higher sheet resistance (R_sh_). The excellent electrical properties of TCO minimize the ohmic losses along the carrier transport and collection pathways at the interface, thereby enhancing the *FF* and overall device efficiency [51, 63].

**TABLE 3:** Diode parameters, N_a_ from *C–V* curves, W_d_ values corresponding to the kesterite‐based TFSCs with various ZnO‐based wide *E*_g_ TCOs.

Sample codes	G_sh_ ^a^ (mS/cm^2^)	*R_s_* ^a^ (Ω cm)	*J*_o_ ^a^ (mA/cm^2^)	A ^b^	W_d_ ^c^ (nm)	N_a_ ^c^ (cm^−3^)
AZO	0.0651	5.88	0.0039	1.30	68.2	2.09 × 10^17^
MGZO	0.0437	3.07	0.0021	1.31	92.8	1.14 × 10^17^
MAZO	0.0605	8.04	0.0037	1.37	71.9	2.04 × 10^17^
MIZO	0.0771	15.01	0.0047	1.49	60.7	3.39 × 10^15^

^a^ Diode parameters were estimated from the data presented in Figure 6a, corresponding to the optimum performance of each sample under dark conditions;

^b^ A was calculated using two data points between 0.4 and 0.5 V in order to minimize the influence of the R_s_ and R_sh_;

^c^ W_d_ and N_a_ were calculated from the data presented Figure 6f, corresponding to the optimum performance of each sample.

As shown in Figure 6d and Table 3, *J*_0_ decreased in the following order: MGZO, MAZO, AZO, and MIZO, with values of 0.0021, 0.0037, 0.0039, and 0.0047 mA/cm^2^, respectively. The TFSC device employing MGZO exhibited a *J*_0_ of nearly half that of the devices using other TCOs. This improvement can be attributed to the wider *E*_g_ and more favorable band alignment, which suppressed interfacial recombination [64]. To further investigate the recombination mechanisms, the diode ideality factor for each device was extracted from the slope of the d*V*/d*J* vs. (*J*+*J*_sc_)^−1^ plot (See Figure 6c) and summarized in Table 3. The A values for all devices were similar, ranging from 1.30 to 1.49. Unlike monocrystalline Si solar cells, the ideality factor in polycrystalline TFSCs, such as kesterite, is strongly influenced by complex phenomena, including tunneling‐enhanced recombination and voltage‐dependent carrier collection [65, 66]. Therefore, interpreting the ideality factor as a direct and isolated metric of the recombination velocity at the *i*‐ZnO/TCO interface has inherent limitations. Nevertheless, the similarity of the ideality factor values across the different TCO‐based devices strongly corroborates that the dominant recombination pathway determining the overall diode characteristics is not governed by the specific TCO material but is instead primarily dominated by the CZTSSe bulk and the primary CZTSSe/CdS interface.

The C‐V plots for each device are shown in Figure 6e, and the extracted N_a_ and W_d_ values are presented in Figure 6f. W_d_ is associated with the drift collection of the photogenerated carriers and interfacial recombination [67]. Notably, the device employing MGZO exhibited a W_d_ of 92.8 nm, an increase of 32.1 nm relative to that using MIZO, which showed the lowest value (60.7 nm). Because all the kesterite absorber layers were fabricated under identical conditions, the observed variation in W_d_ was primarily attributed to differences in the overall built‐in potential (V_bi_) of the device [21, 68]. V_bi_ is fundamentally determined by the Fermi level difference between the *p*‐type CZTSSe absorber and *n*‐type TCO front contact [69, 70]. Based on the UPS analysis (Table S3), MGZO possesses the highest conduction band minimum (−4.06 eV). Owing to its exceptionally high carrier concentration, the Fermi level of MGZO shifts upward, thereby minimizing its work function. This upward shift in the Fermi level of the TCO inherently increased the total V_bi_ across the heterojunction. According to the depletion approximation (*i.e*., $W_{d}\;\propto \;\sqrt{V_{bi}}$) [71], this increased V_bi_ directly results in a wider depletion region extending deeper into the kesterite absorber. Conversely, TCOs with lower CBM levels and lower carrier concentrations, such as MIZO (−4.16 eV), yield a smaller V_bi_ and a correspondingly narrower W_d_. This interpretation is consistent with the observed band alignments and the discussion above.

### 2.6 Comparison for Kesterite TFSCs With MGZO and AZO as TCO Layers

**FIGURE 7:**
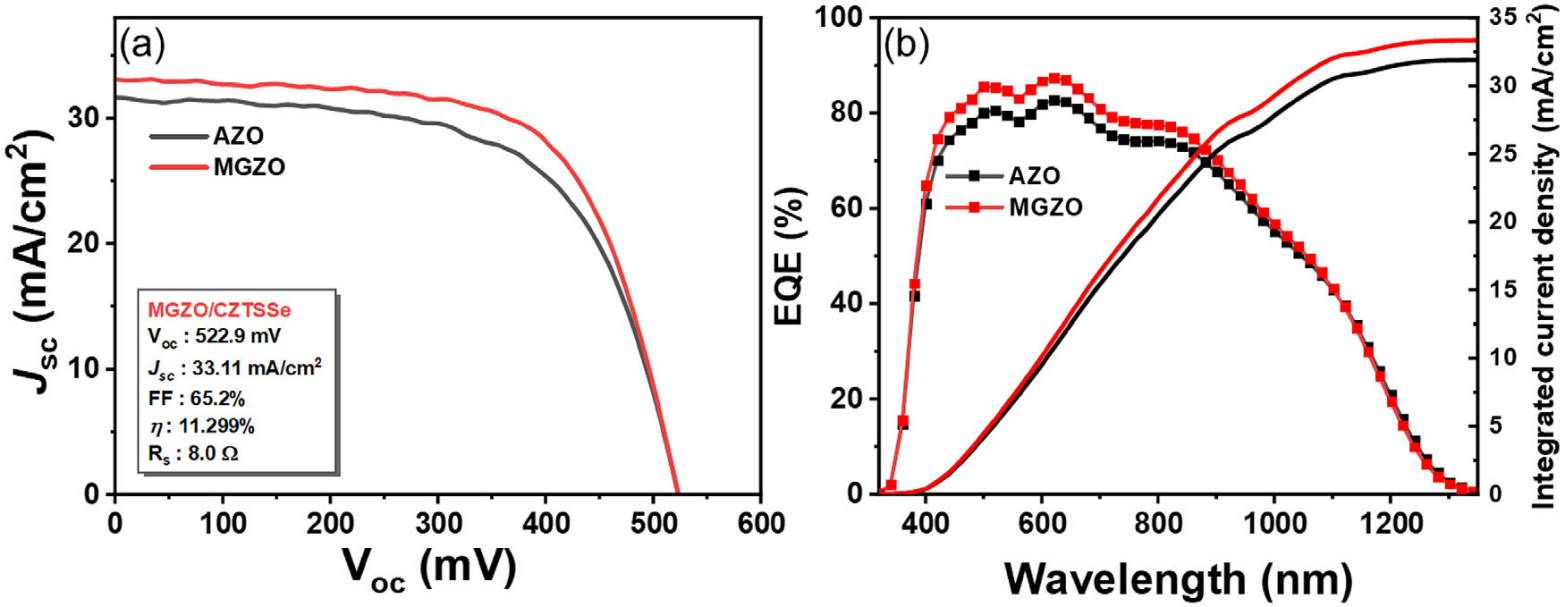
(a) *J–V* curves and (b) EQE spectra and integrated current densities of kesterite TFSC with MGZO and AZO TCOs.

Figure 7 shows the *J–V* curves and EQE spectra of kesterite TFSCs employing MGZO and AZO TCOs. The kesterite TFSC with MGZO device achieved V_oc_ of 522.0 mV, *J*_sc_ of 33.1 mA/cm^2^, *FF* of 65.2%, and PCE of 11.30%. Notably, the kesterite TFSC employing MGZO exhibited a higher EQE up to 900 nm. The observed increase in the EQE spectrum can be attributed to the synergistic effects. First, the enhancement in the short‐wavelength region below 420 nm arises from efficient photon harvesting enabled by the wider *E*_g_ and improved transmittance of the MGZO TCO [7]. Second, the enhancement of the EQE in the long‐wavelength region (420−900 nm) for the MGZO‐based device warrants a rigorous physical explanation, as low‐energy photons penetrate deeper and predominantly generate electron‐hole pairs deep within the *p*‐type CZTSSe absorber layer. The collection efficiency of these deep‐generated carriers is strongly dependent on the effective collection length, which is the sum of W_d_ and the diffusion length (L_d_). Because all the CZTSSe absorber layers were fabricated under strictly identical conditions, the intrinsic bulk minority carrier lifetime and L_d_ were assumed to be constant across all the TFSC devices with different TCOs [72]. Therefore, the substantial improvement in the long‐wavelength carrier collection can be primarily attributed to the extended W_d_. As evidenced by the C‐V profile (Figure 6f), the favorable band alignment and enhanced V_bi_ induced by MGZO as a TCO can maximally lead to a large W_d_ toward the deep absorber region. This physically widened the charge‐carrier collection region. Consequently, the photogenerated electrons in the deep absorber, which would otherwise be lost to bulk recombination during diffusion into each electrode, were swept by the extended built‐in electric field, which directly translated to the observed enhancement in the long‐wavelength EQE response [73].

Substituting the TCO layer simultaneously modifies multiple optical and electrical parameters, making it challenging to isolate a single dominant factor responsible for the observed *J_sc_* and *FF* enhancements. However, their respective contributions can be phenomenologically delineated by examining the specific device responses. The optical gain is primarily evident in the short‐wavelength region (λ < 400 nm) of the EQE spectrum, where the widened optical E_g_ of MGZO significantly reduces the parasitic absorption, providing a direct boost to *J_sc_*. By contrast, electrical and interfacial improvements stemming from superior carrier mobility and favorable band alignment predominantly manifested as minimized transport losses, leading to a substantial reduction in *R*_s_ and a corresponding improvement in *FF*. The complexity of this interplay is further highlighted by the MAZO device; despite its inferior mobility compared to that of AZO, its high optical transmittance partially compensates for electrical losses, resulting in a comparable overall PCE. Therefore, rather than attributing the superior performance of MGZO to a single parameter, we conclude that it is the synergistic outcome of an optimally balanced system in which minimized optical parasitic losses and highly efficient charge transport coexist. Overall, this study provides a clear and systematic insight into the critical roles of ZnO‐based wide *E*_g_ TCOs in governing charge transport, interfacial band alignment, and device performance in kesterite TFSCs. The demonstrated effectiveness of TCO engineering in enhancing current collection, suppressing recombination, and improving the overall device performance highlights its importance for device optimization. Extending this approach to other TFSCs, such as inorganic, organic, and perovskite solar cells, is expected to accelerate their development for practical and industrial applications.

## 3 Conclusion

In this study, the optoelectronic properties of quaternary ZnO‐based wide *E*_g_ TCOs were comprehensively investigated along with their impact on the device parameters and performance of kesterite TFSCs. Wide *E*_g_ TCOs reduce optical losses in the UV–vis region, enable more efficient photon harvesting, and promote a favorable band alignment. In addition, TCOs with superior electrical properties suppress interfacial recombination and enhance carrier collection, leading to improved TFSC device performance. Among the investigated ZnO‐based wide *E*_g_ TCOs, MGZO thin film exhibited the widest *E*_g_ of 3.82 eV and excellent electrical properties (8.78 × 10^20^ cm^−3^ of carrier concentration and 24 cm^2^/Vs of mobility). It should be noted that the performance enhancement of MGZO‐based TFSCs cannot be exclusively attributed to isolated parameters such as the BM effect or intrinsic carrier mobility. Substituting the TCO layer simultaneously alters multiple interrelated factors, including the crystallinity, surface roughness, morphology of the thin film, and potential interfacial damage induced during sputtering deposition, such that the overall device performance is inherently governed by their complex interplay. Furthermore, although our dark *J*‐V and C‐V analyses indicated improved diode characteristics and an extended W_d_, the direct quantification of suppressed interfacial recombination requires further advanced transient or spectroscopic characterization tools. Therefore, rather than claiming a single definitive mechanism, we conservatively suggest that the superior performance of the TFSC device with the MGZO TCO is a correlative and synergistic outcome. The structurally improved smooth morphology widened the optical transmission and favorable interfacial energetics collectively contributed to mitigating transport losses and reducing recombination pathways, thereby leading to enhanced *J_sc_* and *FF*. As a result, the TFSC employing MGZO as the TCO layer achieved a high *J*_sc_ of 33.1 mA/cm^2^ and a PCE of 11.30%. The application of quaternary TCO with a wide *E*_g_, high transmittance, and excellent electrical properties in kesterite TFSC devices synergistically enhances the device parameters and performance. These findings can easily be extended to other TFSCs and optoelectronic devices.

## 4 Experimental Sections

To fabricate the AZO, MGZO, MAZO, and MIZO sputtering targets, Al_2_O_3_ (99.99%), MgO (99.99%), Ga_2_O_3_ (99.99%), In_2_O_3_ (99.99%), and ZnO (99.99%) powders were prepared. Each sputtering target was prepared with multiple compositions, and the optimal weight percent formulations were as follows: Al_0.020_‐ZnO_0.980_, Mg_0_._010_Ga_0.018_‐ZnO_0.972_, Mg_0.030_Al_0.020_‐ZnO_0.950_, and Mg_0.030_In_0.030_‐ZnO_0.940_. The targets were fabricated using conventional solid‐state reaction methods, in which the constituent powders were mechanically mixed and subsequently subjected to a high‐temperature diffusion reaction. First, separate precursor mixtures were prepared: (i) Al_2_O_3_ and ZnO; (ii) Ga_2_O_3_, MgO, and ZnO; (iii) MgO, Al_2_O_3_, and ZnO; and (iv) MgO, In_2_O_3_, and ZnO. Each mixture was uniformly mixed via ball milling in Nalgene bottles containing ethanol and zirconia balls for 100 h. The slurries were then dried in an oven for 72 h and pulverized into fine powder. The powders were compacted by cold isostatic pressing into 2‐inch circular targets. The first compaction was conducted at 180 kg cm^−2^, followed by a second compaction under hydrostatic conditions at 360 kg cm^−2^ using a mold. Finally, the compacts were sintered at 1100°C for 4 h to obtain sputtering targets.

Soda‐lime glass (SLG) substrates were cleaned sequentially in isopropyl alcohol, ethanol, and distilled water for 10 min each. The cleaned SLG substrates were then used to deposit AZO, MGZO, MAZO, and MIZO thin films via radio‐frequency (RF) magnetron sputtering. During deposition, an RF power density of 1.97 W cm^−2^, a working pressure of 5 mTorr, and a substrate temperature of 200°C were maintained under a continuous Ar flow. The thickness of all thin films was maintained at 650 nm. The detailed processes for fabricating kesterite TFSCs are described in our previous papers [74, 75, 76, 77]. Briefly, (i) elemental Zn, Sn, and Cu were sequentially deposited by direct current (DC) magnetron sputtering onto Mo‐coated SLG substrates to form Cu/Sn/Zn stacks on Mo; (ii) the stacked metallic precursors were preannealed at 280°C for 1 h; (iii) the preannealed precursors, placed with S and Se powders in a graphite box, were subjected to rapid thermal annealing at 530°C for 8.5 min; (iv) an *n*‐type CdS buffer layer serving as the *p‐n* junction partner and buffer was deposited on the CZTSSe absorber by chemical bath deposition; (v) TCO stacks of *i*‐ZnO/ZnO‐based compounds including AZO, MGZO, MAZO, or MIZO were deposited by RF magnetron sputtering; and (vi) a mask‐defined Al grid as the top electrode was deposited onto the TCO by DC sputtering.

## Conflicts of Interest

The authors declare no conflicts of interest.

## Supporting information




**Supporting File**: smll74042‐sup‐0001‐SuppMat.docx.

## Data Availability

The data that supports the findings of this study are available in the supplementary material of this article.
