# Supplementary material for: Insight into the Impact of Wide Bandgap Transparent Conducting Oxide on the Performance of Thin Film Solar Cells
Source: Small. 2026 Jun 9;22(41):e74042. doi: 10.1002/smll.74042 (PMC13392782; doi:10.1002/smll.74042)
Supplement: Supplementary file 1 — Supporting File: smll74042‐sup‐0001‐SuppMat.docx. [file SMLL-22-e74042-s001.docx]

**Supporting information**

**Insight into the impact of wide band gap transparent conducting oxide on the performance of thin film solar cells**

*Youseong Park^1^, Jun Sung Jang^1^, Dhanaji B. Malavekar^1^, Hojun Choi^1^, Do Hyun Kim ^1^, Dong Hyun Kang^1^, Donghoon Song^2^, Seung Wook Shin^3,^* and Jin Hyeok Kim^1,^**

^1^Department of Materials Science and Engineering and Optoelectronics Convergence Research Center, Chonnam National University, Gwangju, 61186, Republic of Korea

^2^Department of Chemical Engineering, Sunchon National University, Suncheon, 57922, Republic of Korea

^3^Future Agricultural Research Division, Rural Research Institute, Korea Rural Community Corporation, 870 Haean-ro Sangnok-gu, Ansan-si, Gyeonggi-do, Republic of Korea.

*Corresponding authors E-mail

Seung Wook Shin: swshin1211@gmail.com

Jin Hyeok Kim: jinhyeok@chonnam.ac.kr

**Material characterizations**

An X-ray diffractometer (X’Pert PRO, PANALYTICAL, UK, operating at 20 kV with Cu kα radiations) was utilized to analyze the structural characteristics of the TCO and kesterite thin films. The surface morphology and roughness of the thin films were observed and analyzed using field-emission scanning electron microscopy (FE-SEM S 4700, Hitachi, Japan) and atomic force microscopy (AFM; Dimension Edge, Bruker, Germany). The electrical properties of the thin films were analyzed using a Hall measurement system (HMS-3000, Ecopia, Korea) at room temperature (300 K) and at a low temperature (77 K). Sheet resistance (R_sh_) was measured using the four-point probe method. The transmittance, bandgap energy (E_g_), and absorption edge of the thin films were determined using a UV-vis spectrophotometer (Cary 100, Varian, Australia) at room temperature. The power conversion efficiency and external quantum efficiency of the kesterite TFSCs were characterized using a solar simulator (WXS-1552-L2, Wacom, Japan) and a quantum efficiency measurement system CEP-25BX (Buncou Keiki Co. Ltd., CEP-25BX, Japan). A Keithley 4200A-SCS was used to investigate the leakage current and characteristics of the TFSC devices. A Raman spectrometer (Tokyo Instruments, FLEX G, Energy Convergence Core Facility, Chonnam, South Korea) was used to analyze the structural characteristics of the kesterite TFSC devices.

**Figure of merit (FOM)**

The FOM is calculated using the one-diode model according to Eq. (S1) and (S2).

$T={(1+\frac{377}{{2R}_{s}}\frac{\sigma_{OP}}{\sigma_{DC}})}^{-2}$, (S1)

where T is the transmittance, R_sh_ is the sheet resistance, *σ_op_* is the DC conductivity, *σ_dc_* is the optical conductivity, and 377 is the free-space impedance^[1]^.

$\frac{\sigma_{DC}}{\sigma_{OP}}=\frac{377}{{2R}_{sh}(T^{-\frac{1}{2}}-1)}$ (S2)

**Dark current characterization**

The device parameters, including G_sh_, *J*_o_, and *R*_s_, for the kesterite TFSCs listed in **Table 2** were calculated using the one-diode model according to Eq. (S3)^[2]^.

𝐽 ‒ 𝐽_sc_ = 𝐽_0_ exp [ 𝑞(𝑉 ‒ 𝑅_𝑠_𝐽)/𝐴𝑘𝑇]+/𝐺_sh_, (S3)

where *J*_sc_ is the photocurrent density; G_sh_ is the shunt conductance; A is the ideal diode factor; *J*_o_ is the saturation current density; *R*_s_ is the series resistance; *q* is the electronic charge; and *k* is the Boltzmann constant. The derivative d*J*/dV *vs.* *(J+J_sc_)*^−1^ is plotted (**Fig. 6c**). A linear fit to the data yields an intercept of *R*_s_ and a slope of A*k*T/*q* in **Fig. 6c**.

**Capacitance characterization**

The room temperature capacitance (C) *vs.* voltage (V) plot of the solar cell devices was collected from -1.5 to 1 V DC bias voltage using 25 mV and 100 kHz alternating current (AC) signal. An impedance analyzer (Hewlett Packard, HP4284) was used to perform the C-V measurements.

The junction depletion width (W) was extracted from the *C-V* plot shown in **Fig. 6f** using Eq. (S4)^[2]^.

$C=\frac{\varepsilon_{0}\varepsilon_{r}A}{w}$, (S4)

where ε_0_ is free space permittivity, and ε_r_ is the relative dielectric constant of kesterite. The value of C is obtained from **Fig. 6e** at V = 0. The ε_r_ value of 8.2 is obtained from published papers. The area (A) of the TFSC is 3 × 10^−5^ m^2^. Subsequently, the free carrier concentration (N_a_) was calculated using Eq. (S5).

$\boldsymbol{N=}\frac{\boldsymbol{-2}}{\boldsymbol{q}\boldsymbol{\varepsilon}_{\boldsymbol{0}}\boldsymbol{\varepsilon}_{\boldsymbol{\gamma}}\boldsymbol{A}^{\boldsymbol{2}}}\left[ \frac{d\left( C^{-2} \right)}{ⅆv} \right]^{-1}$**,** (S5)

where *q* denotes the electronic charge. The value for [*d*(C^−2^)/dV)]^−1^ is taken from the slope of (1/C^2^)–V.

**Table S1.** Reported *E*_g_ of ZnO-based materials in the literature.

| Sample | Deposition technologies | Thickness(nm) | Eg (eV) |
| --- | --- | --- | --- |
| **ZnO^[3]^** | AACVD  (Aerosol-Assisted Chemical Vapor Deposition) | **760** | **3.14** |
| **ZnO:Ga(0.4)^[3]^** | AACVD | **410** | **3.19** |
| **ZnO:Ga(0.4)^[3]^** | AACVD | **490** | **3.27** |
| **ZnO:Ga(2.3)^[3]^** | AACVD | **720** | **3.39** |
| **ZnO:Ga(3.0)^[3]^** | AACVD | **480** | **3.40** |
| **ZnO:Ga(4.3)^[3]^** | AACVD | **510** | **3.42** |
| **ZnO:Ga(6.1)^[3]^** | AACVD | **490** | **3.37** |
| **ZnO:Al(3.1)^[4]^** | Spin coating | **551** | **3.31** |
| **ZnO:Al(6.2)^[4]^** | Spin coating | **638** | **3.38** |
| **ZnO:Al(9.4)^[4]^** | Spin coating | **880** | **3.45** |
| **ZnO:Mg(3)^[5]^** | Electrodeposition | **400** | **3.38** |
| **ZnO:Mg(4)^[5]^** | Electrodeposition | **400** | **3.40** |
| **ZnO:Mg(5)^[5]^** | Electrodeposition | **400** | **3.41** |
| **ZnO:Mg(7)^[6]^** | CVD  (Chemical Vapor Deposition) | **.** | **~3.55** |
| **ZnO:Mg(12)^[6]^** | CVD | **.** | **~3.65** |
| **ZnO:Mg(18)^[6]^** | CVD | **.** | **~3.8** |
| **ZnO:Mg(25)^[6]^** | CVD | **.** | **3.96** |
| **ZnO:Mg(31)^[6]^** | CVD | **.** | **4.10** |
| **ZnO:In(1)^[7]^** | RF sputtering | **819** | **3.39** |
| **ZnO:In(3)^[7]^** | RF sputtering | **977** | **3.40** |
| **ZnO:In(5)^[7]^** | RF sputtering | **643** | **3.46** |
| **ZnO:In(7)^[7]^** | RF sputtering | **745** | **3.32** |
| **ZnO:In(9)^[7]^** | RF sputtering | **704** | **3.21** |

**
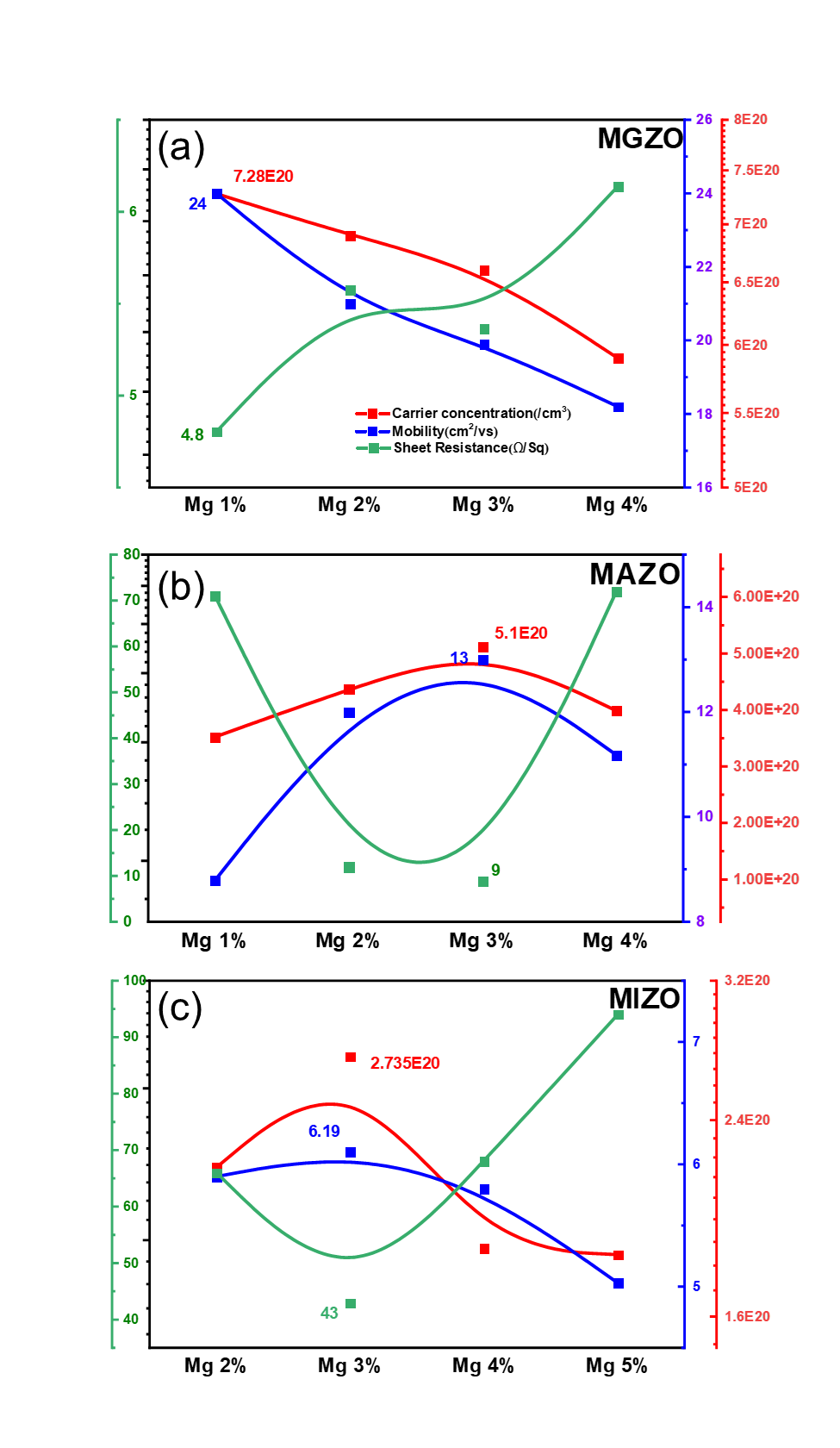
**

Fig. S1**.** Electrical Properties of (a) MGZO, (b) MAZO, and (c) MIZO as a function of Mg contents.

**
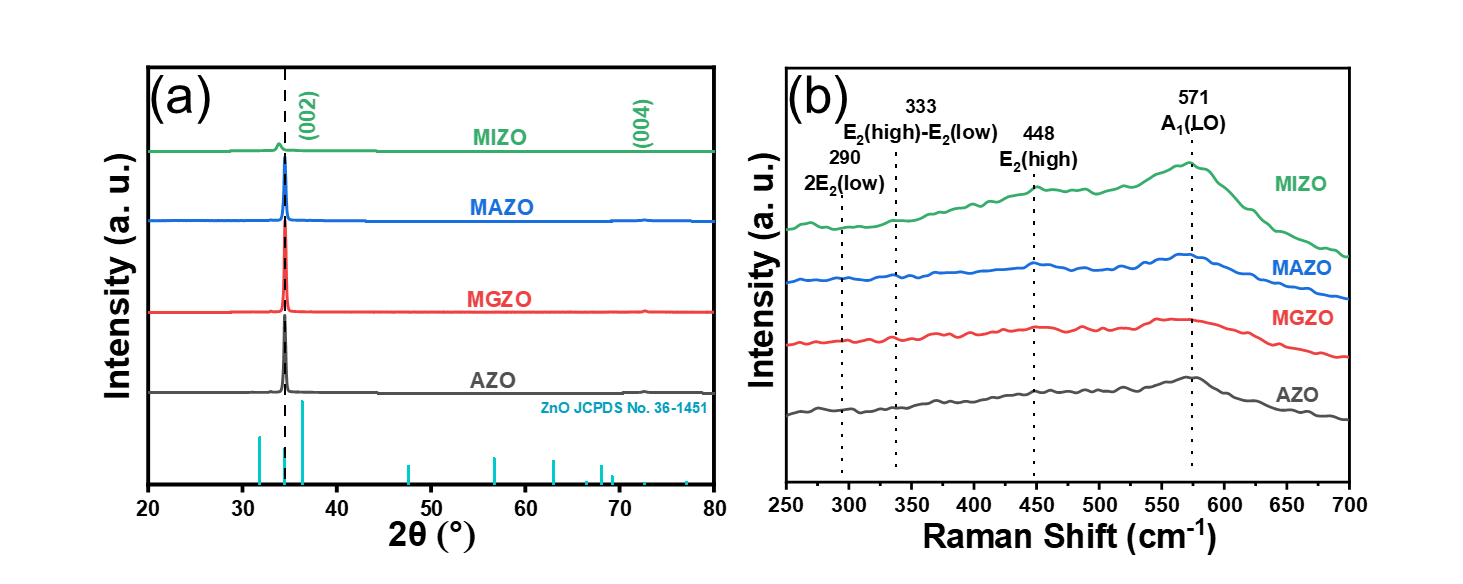
**

Fig. S2**.** (a) X-ray diffraction patterns, (b) Raman spectra of different ZnO-based wide *E*_g_ TCOs.

**Note S1.**

**Fig. S1 (**a) shows the XRD patterns of the different ZnO-based wide *E*_g_ TCO materials. All the samples grew preferentially along the (002) and (004) planes, which are the main peaks of the wurtzite-structured ZnO crystal system^[8]^. In addition, no distinct diffraction peaks attributable to the dopant elements (Mg, Ga, In, or Al) or related oxide compounds were detected, indicating that the dopant elements were well-incorporated into the ZnO lattice. MIZO exhibits peaks located at slightly lower angles and peak intensities than the other samples. The reduced peak intensity of MIZO may be attributed to In-induced lattice distortion. The larger ionic radius of In relative to Zn can introduce lattice strain in ZnO, leading to weakened diffraction peaks and reduced crystallinity^[9,10]^. **Fig. S2 (b)** shows the Raman spectra of the TCO thin films. In general, ZnO-based materials tend to exhibit variations in the intensities of their characteristic peaks depending on the dopant species and concentration, and the peak observed near 571 cm^−1^ is commonly attributed to lattice mismatch and defect-related vibrations induced by dopant incorporation^[11]^. Among the investigated TCO materials, MGZO exhibits the lowest peak intensity in this region, indicating its superior crystallinity.


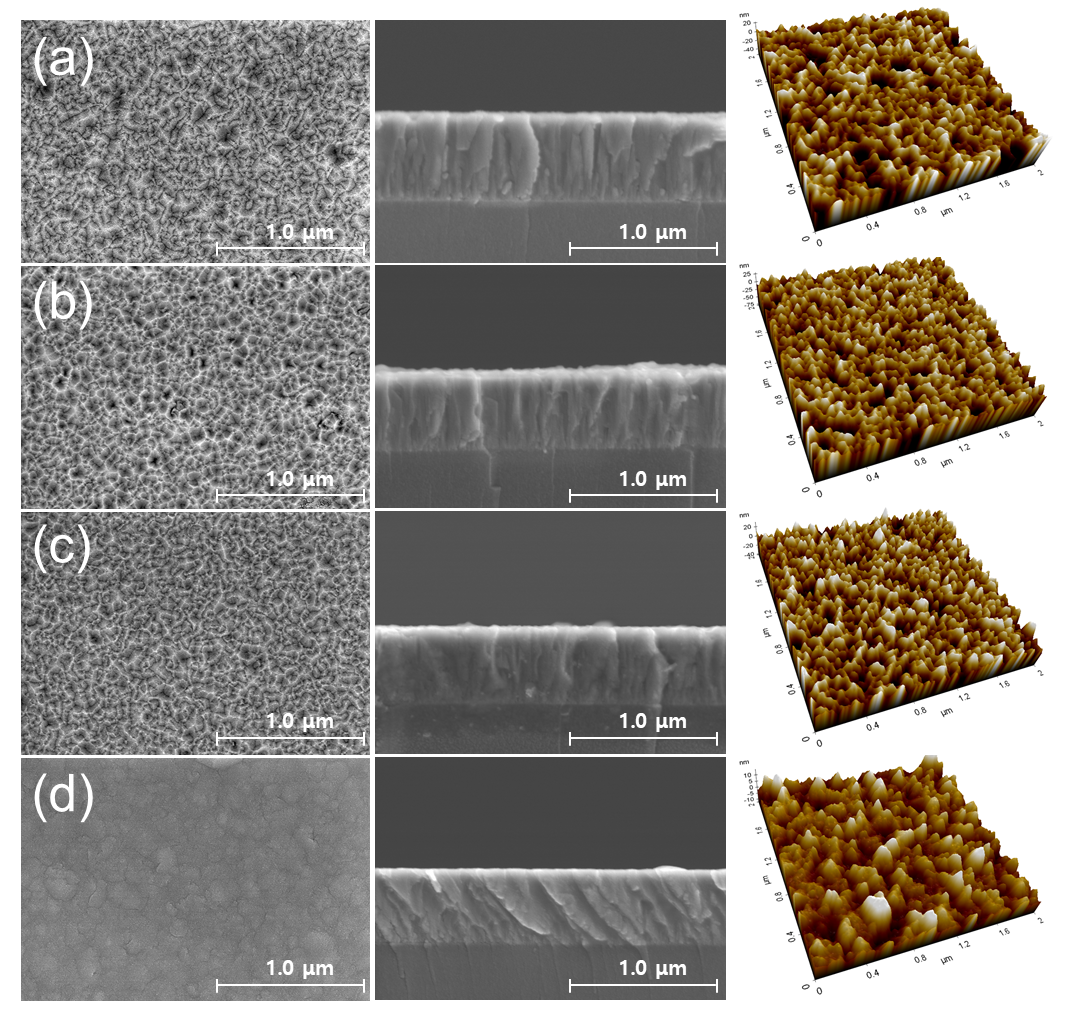
Fig. S3**.** FE-SEM and AFM images of ZnO-based wide *E*_g_ TCOs; (a) AZO, (b) MGZO, (c) MAZO and (d) MIZO, respectively.

**Note S2.**

All the ZnO-based wide *E*_g_ TCO thin films were deposited at a thickness of 650 nm. Cross-sectional SEM images show that AZO, MGZO, and MAZO grew preferentially along the *c*-axis (002) plane, whereas MIZO grew along the inclined plane. The growth direction of the MIZO is consistent with the XRD pattern. AFM measurements were used to quantify the surface roughness of the deposited thin films in terms of arithmetic average roughness (R_a_) and root-mean-square roughness (RMS). A smoother surface is generally beneficial because it reduces surface scattering and facilitates charge transport^[12,13]^. Among the samples, MGZO exhibited the lowest R_a_ and RMS roughness values, and the roughness increased sequentially in the following order: AZO, MAZO, and MIZO.

**Table S2.** R_a_ and RMS values calculated from AFM images for ZnO-based wide E_g_ TCOs.

| **Sample** | **R_a_ (nm)** | **RMS (nm)** |
| --- | --- | --- |
| AZO | 6.47 | 8.25 |
| MGZO | 5.99 | 7.93 |
| MAZO | 9.07 | 12.14 |
| MIZO | 2.84 | 3.62 |


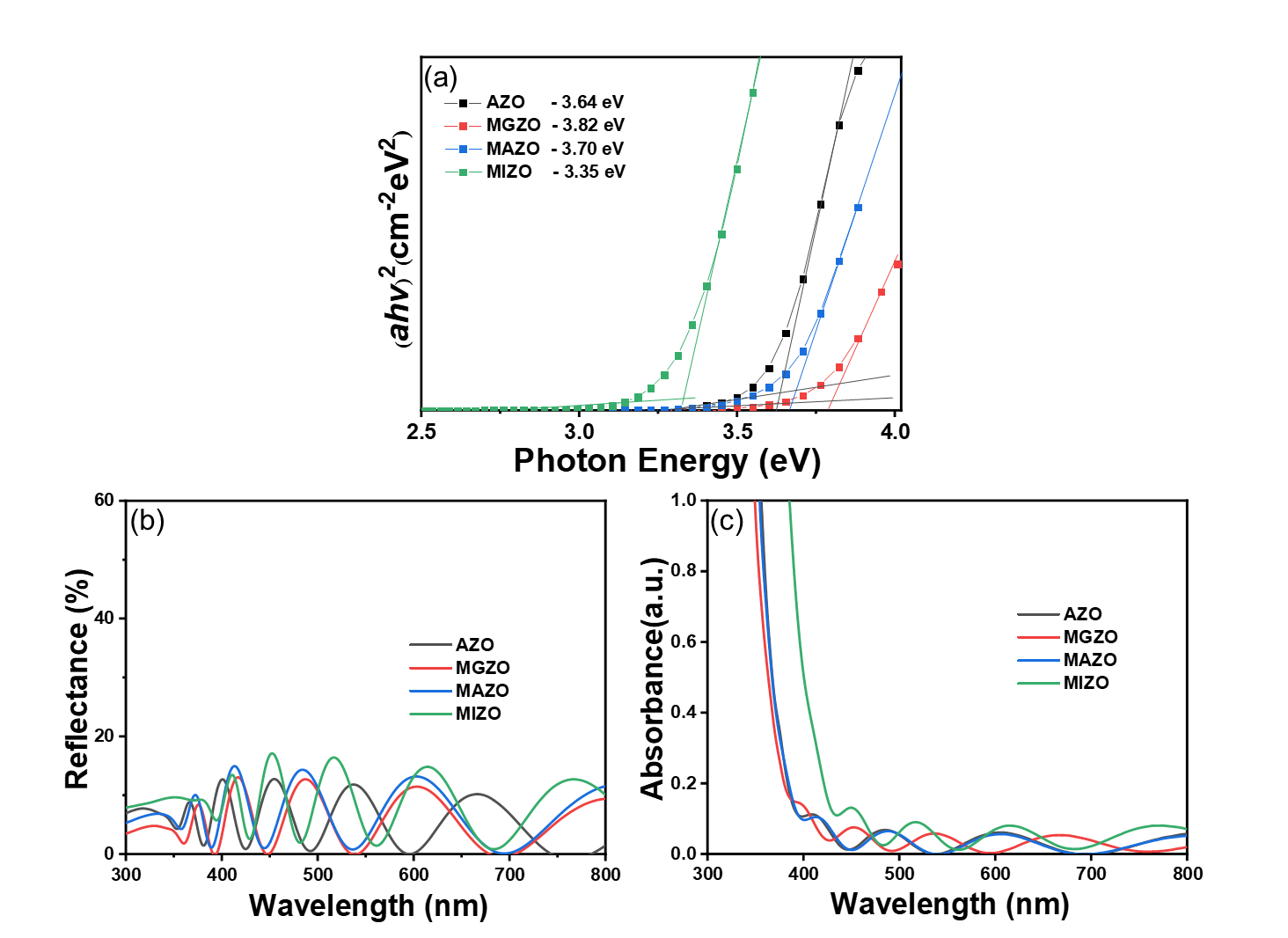


Fig. S4**.** (a) *E*_g_ calculated from Tauc-plot equation, (b) reflectance, and (c) absorbance of different ZnO-based wide *E*_g_ TCOs.

**Note S3.**

*E*_g_ was determined from

${(\alpha h\nu)}^{n}=C\left( h\nu-E_{g} \right)$ (S3)

where $\alpha$ is the absorption coefficient, $h\nu$ is the photon energy, and $n$ is the exponent defined by the electronic transition type, for example, $n=\frac{1}{2}$ for direct allowed transitions and $n=2$ for indirect allowed transitions^[14]^. The optical *E*_g_ was extracted from the Tauc plots, and a systematic blue shift of the absorption edge was observed, indicating improved transmission at short wavelengths. This trend agrees with the transmittance behavior and the corresponding E_g_ values shown in **Fig. S4(a)**. Among the samples, MGZO exhibits the widest optical *E*_g_ of 3.82 eV. As shown in **Fig. S4(b) and (c)**, both the absorptance and reflectance decreased monotonically in the order MIZO > AZO > MAZO > MGZO. Because parasitic optical losses in the window/TCO stack are particularly relevant in the UV-visible region (300–800 nm) for CZTSSe TFSCs, the combination of higher transmittance, lower absorptance and reflectance, and wider *E*_g_ suggests that these TCOs, when employed as window layers, can increase the number of photons reaching the absorber while minimizing optical losses.

**Table S3.** Quantified values of the E_g_, Burstein-Moss (BM) shift, and E_g_ renormalization (BGR) for each thin film.

| Sample | E_BM_ | E_BGR_ | Net shift |
| --- | --- | --- | --- |
| AZO | 0.706 | 0.306 | 0.40 |
| MGZO | 1.026 | 0.506 | 0.52 |
| MAZO | 0.874 | 0.444 | 0.43 |
| MIZO | 0.544 | 0.344 | 0.20 |


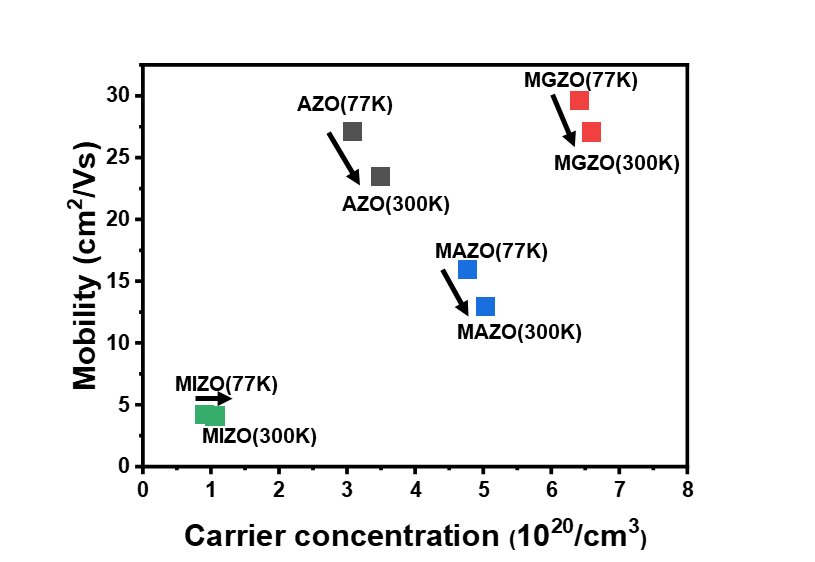


**Fig. S5**. Hall measurement results of each TCO thin film obtained at 77 and 300 K.


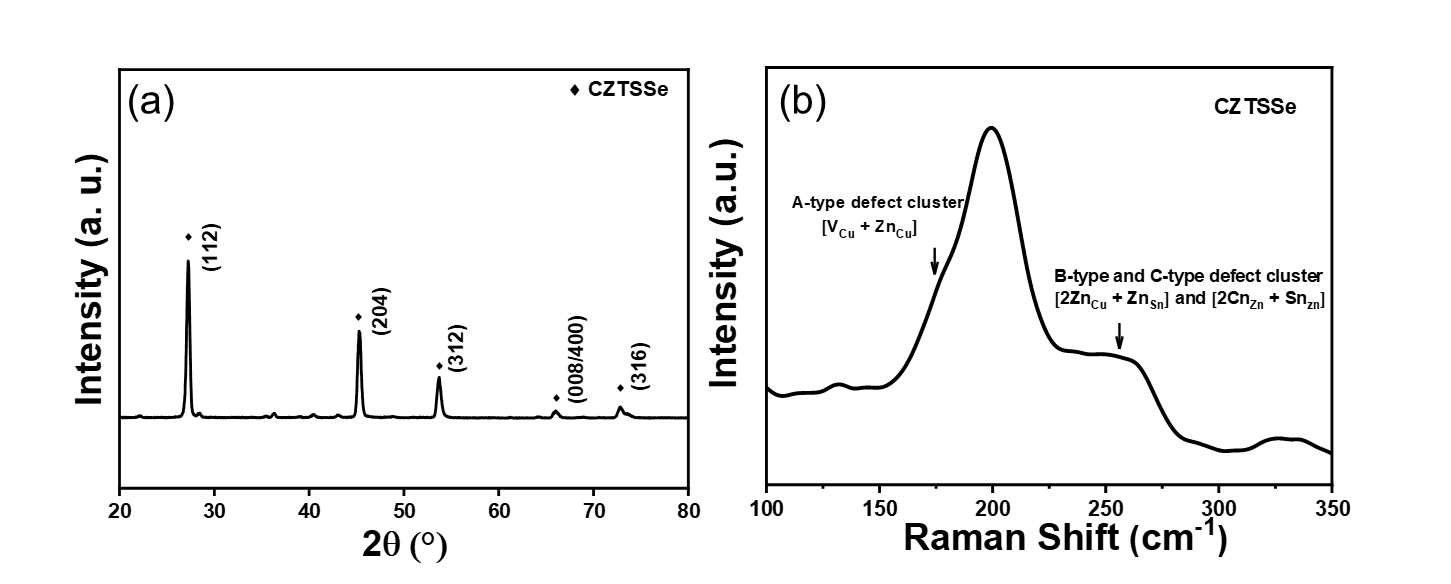


Fig. S6**.** (a) X-ray diffraction pattern and (b) Raman spectrum of kesterite sample.

Fig. S7**.** Survey XPS spectrum of kesterite sample.


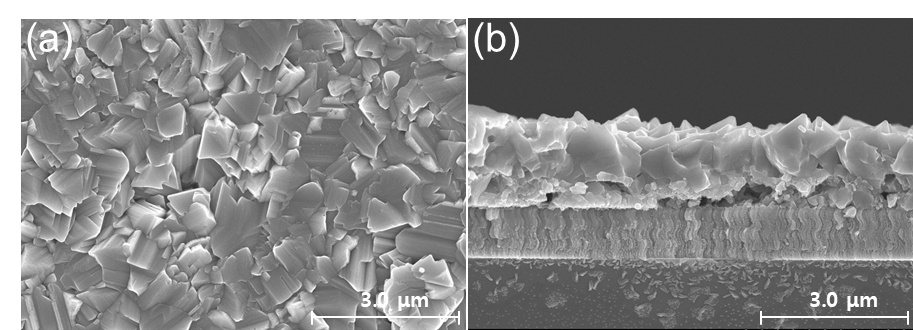


Fig. S8**.** (a) Surface and (b) cross-sectional FE-SEM images for the kesterite sample.

**Note S4.**

X-ray diffraction, Raman spectroscopy, X-ray photoelectron spectroscopy, and scanning electron microscopy were performed to examine the crystallographic characteristics and microstructure of the kesterite absorber. As shown in **Fig. S6(a)**, the XRD pattern exhibited pronounced diffraction peaks at approximately 27.5°, 45.6°, and 53°, which can be indexed to the (112), (220), and (312) planes of the kesterite phase, respectively^[15]^. These results indicate that the kesterite absorber employed in this study was well-formed as a high-quality thin film. **Fig. S6(b)** shows the Raman spectrum and demonstrates the defects and related defect cluster information. A strong Raman peak associated with the kesterite phase was observed at 198 cm^-1^, while a weak peak at 172 cm^-1^ corresponds to A-type [V_Cu_+Zn_Cu_] defects. In addition, peaks appearing at 233 cm^-1^ were attributed to B-[2Zn_Cu_+Zn_Sn_] and C-type [2Cu_Zn_+Sn_Zn_] defect clusters^[16]^. A detailed discussion and deeper insight into the CZTSSe absorber and the related defect chemistry are provided in our previous studies^[17,18]^. XPS analysis was conducted to evaluate the ionic and chemical states of the CZTSSe absorbers (**Fig. S7**). Distinct core-level peaks corresponding to Cu, Zn, Sn, S, and Se are clearly observed, confirming the successful synthesis of the CZTSSe absorber**. Fig. S8** shows FE-SEM images of the CZTSSe film, revealing densely packed, slightly faceted, and rounded grains characteristic of CZTSSe on the surface. The thickness of the CZTSSe absorber is approximately 1.5 μm.

**
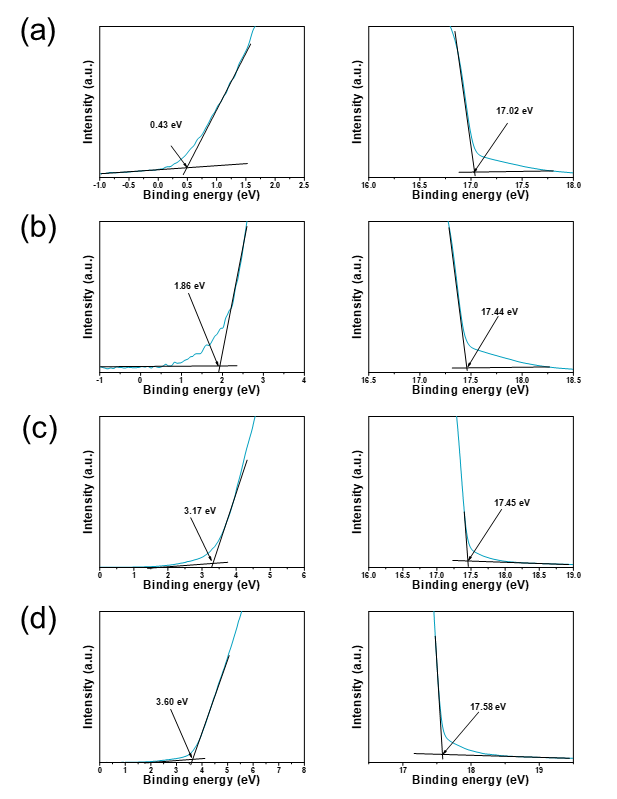
**

**
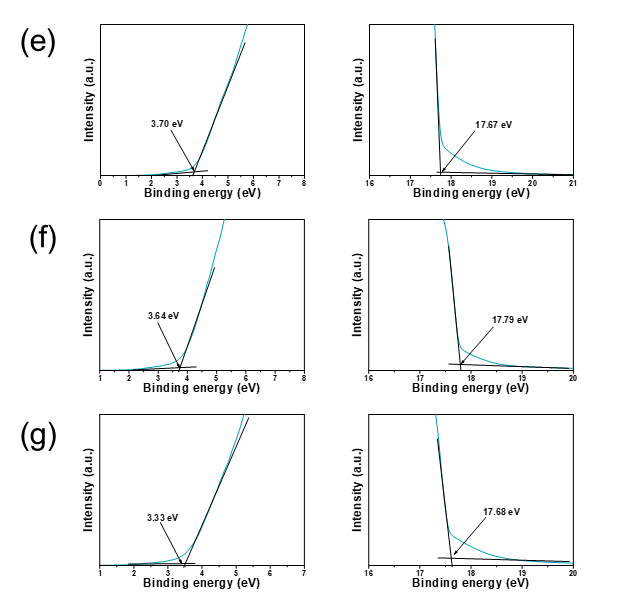
**

**Fig. S9.** Secondary electron cutoff edge and the Fermi edge of the CZTSSe device from UPS spectra; (a) CZTSSe, (b) CZTSSe/CdS, (c) CZTSSe/CdS/i-ZnO, (d) CZTSSe/CdS/i-ZnO/AZO, (e) CZTSSe/CdS/i-ZnO/MGZO, (f) CZTSSe/CdS/i-ZnO/MAZO, and (g) CZTSSe/CdS/i-ZnO/MIZO, respectively.

**Reference**

[1] J. Kim, J. S. Jang, S. W. Shin, H. Park, W. L. Jeong, S. H. Mun, J. H. Min, J. Ma, J. Heo, D. S. Lee, J. J. Woo, J. H. Kim, H. J. Kim, *Small* **2023**, *19*, 2207966.

[2] M. C. Baek, J. S. Jang, V. C. Karade, M. P. Suryawanshi, J. H. Kim, T. E. Hong, S. W. Park, S. W. Shin, J. H. Kim, *Chem.Eng. J.* **2024**, *479*, 147842.

[3] S. Chen, G. Carraro, D. Barreca, A. Sapelkin, W. Chen, X. Huang, Q. Cheng, F. Zhang, R. Binions, *J. Mater. Chem. A* **2015**, *3*, 13039.

[4] G. El Hallani, M. Khuili, N. Fazouan, A. Liba, H. A. El Makarim, E. H. Atmani, *Chem. Phys. Impact* **2024**, *8*, 100648.

[5] R. Kara, L. Mentar, A. Azizi, *RSC Adv.* **2020**, *10*, 40467.

[6] H. Nishinaka, Y. Kamada, N. Kameyama, S. Fujita, *Phys. Status Solidi B* **2010**, *247*, 1460.

[7] G. C. Xie, L. Fanga, L. P. Peng, G. B. Liu, H. B. Ruan, F. Wu, C. Y. Kong, *Phys. Procedia* **2012**, *32*, 651.

[8] M. F. Malek, M. H. Mamat, M. Z. Musa, Z. Khusaimi, M. Z. Sahdan, A. B. Suriani, A. Ishak, I. Saurdi, S. A. Rahman, M. Rusop, *J. Alloys Compd.* **2014**, *610*, 575.

[9] S. Benzitouni, M. Zaabat, A. Mahdjoub, A. Benaboud, B. Boudine, *Mater. Sci-Pol.* **2018**, *36*, 427.

[10] J. H. Lim, S. M. Lee, H. S. Kim, H. Y. Kim, J. Park, S. B. Jung, G. C. Park, J. Kim, J. Joo, *Sci. Rep.* **2017**, *7*, 41992.

[11] R. Baghdad, B. Kharroubi, A. Abdiche, M. Bousmaha, M. A. Bezzerrouk, A. Zeinert, M. El Marssi, K. Zellama, *Superlatt. Microstruct.* **2012**, *52*, 711.

[12] K. Gu, X. Zhou, Z. Zhang, K. Tang, J. Huang, L. Wang, *Mater. Lett.* **2020**, *278*, 128416.

[13] W. H. Luo, T. K. Tsai, J. C. Yang, W. M. Hsieh, C. H. Hsu, J. S. Fang, *J. Electron. Mater.* **2009**, *38*, 2264.

[14] T. Prasada Rao, M. C. Santhosh Kumar, *J. Alloys Compd.* **2010**, *506*, 788.

[15] Y. T. Hsieh, Q. Han, C. Jiang, T. Bin Song, H. Chen, L. Meng, H. Zhou, Y. Yang, *Adv. Energy Mater.* **2016**, *6*, 1502386.

[16] M. Dimitrievska, F. Oliva, M. Guc, S. Giraldo, E. Saucedo, A. Pérez-Rodríguez, V. Izquierdo-Roca, *J. Mater. Chem. A* **2019**, *7*, 13293.

[17] V. Karade, E. Choi, M. G. Gang, H. Yoo, A. Lokhande, P. Babar, J. S. Jang, J. Seidel, J. S. Yun, J. Park, J. H. Kim, *ACS Appl. Mater. Interfaces* **2021**, *13*, 429.

[18] J. S. Jang, Y. Park, M. He, V. C. Karade, M. C. Baek, D. Song, M. P. Suryawanshi, H. Choi, D. H. Kang, Y. Kim, S. W. Shin, J. H. Kim, *J. Mater. Chem. A* **2025**, *13*, 36741.
